# Molecular identification of different trypanosome species and subspecies in tsetse flies of northern Nigeria

**DOI:** 10.1186/s13071-016-1585-3

**Published:** 2016-05-23

**Authors:** Clement Isaac, Marc Ciosi, Alana Hamilton, Kathleen Maria Scullion, Peter Dede, Igho Benjamin Igbinosa, Oyebiguwa Patrick Goddey Nmorsi, Dan Masiga, C. Michael R. Turner

**Affiliations:** Department of Zoology, Ambrose Alli University, Ekpoma, Nigeria; Institute of Infection Immunity and Inflammation, Sir Graeme Davis Building, University of Glasgow, 120 University Place, Glasgow, G12 0PT UK; International Centre for Insect Physiology and Ecology (ICIPE), P.O. Box 30772, 00100 Nairobi, Kenya; Nigerian Institute of Trypanosomiasis Research (NITR), Kaduna, Nigeria; Department of Medical Microbiology and Parasitology, College of Health Sciences, Delta State University, Abraka, Nigeria

**Keywords:** *Glossina palpalis palpalis*, *Glossina tachinoides*, *Glossina morsitans submorsitans*, Animal African Trypanosomiasis, *Trypanosoma congolense* Savannah *Trypanosoma congolense *Forest*Trypanosoma godfreyi Trypanosoma simiae*, *Trypanosoma vivax*, ITS1

## Abstract

**Background:**

Animal African Trypanosomiasis (AAT) is caused by several species of trypanosomes including *Trypanosoma congolense, T. vivax, T. godfreyi, T. simiae* and *T. brucei*. Two of the subspecies of *T. brucei* also cause Human African Trypanosomiasis. Although some of them can be mechanically transmitted by biting flies; these trypanosomes are all transmitted by tsetse flies which are the cyclical vectors of *Trypanosoma congolense*, *T. godfreyi*, *T. simiae* and *T. brucei*. We present here the first report assessing the prevalence of trypanosomes in tsetse flies in Nigeria using molecular tools.

**Methods:**

488 tsetse flies of three species, *Glossina palpalis palpalis*, *G. tachinoides* and *G. morsitans submorsitans* were collected from Wuya, Niger State and Yankari National Park, Bauchi State in 2012. Trypanosomes were detected and identified using an ITS1 PCR assay on DNA purified from the ‘head plus proboscis’ (H + P) and abdomen (ABD) parts of each fly.

**Results:**

*T. vivax* and *T. congolense* Savannah were the major parasites detected. Trypanosomes prevalence was 7.1 % in *G. p. palpalis*, 11.9 % in *G. tachinoides* and 13.5 % in *G. m. submorsitans*. Prevalences of *T. congolense* Savannah ranged from 2.5 to 6.7 % and of *T. vivax* were approximately 4.5 %. *Trypanosoma congolense* Forest, *T. godfreyi* and *T. simiae* were also detected in the site of Yankari. The main biological and ecological determinants of trypanosome prevalence were the fly sex, with more trypanosomes found in females than males, and the site, with *T. congolense* subspp. being more abundant in Yankari than in Wuya. As expected, the trypanosome species diversity was higher in Yankari National Park than in the more agricultural site of Wuya where vertebrate host species diversity is lower.

**Conclusions:**

Our results show that *T. congolense* Savannah and *T. vivax* are the main species of parasite potentially causing AAT in the two study sites and that Yankari National Park is a potential reservoir of trypanosomes both in terms of parasite abundance and species diversity.

**Electronic supplementary material:**

The online version of this article (doi:10.1186/s13071-016-1585-3) contains supplementary material, which is available to authorized users.

## Background

Tsetse flies are the cyclical vectors of African trypanosomes. Many of these trypanosomes are pathogens of humans and livestock, causing human African trypanosomiasis (HAT) and animal African trypanosomiasis (AAT), diseases with major medical and economic impact. The human disease is caused by two subspecies of *Trypanosoma brucei* while AAT is caused by at least seven species/subspecies of trypanosomes [1]. *Trypanosoma vivax* and *Trypanosoma congolense* Savannah are the most important pathogens causing AAT, because of their predominance throughout sub-Saharan Africa and of their economic impact on animal production [1]. AAT is recognized as an important constraint to agricultural development, causing losses to livestock producers and consumers exceeding $1300 million per year [2]. The number of disease control methods within the vertebrate host is limited. Moreover, the evolution of resistances to trypanocidal drugs makes chemotherapy difficult to sustain for the control of AAT [3]. For these reasons, vector control remains a very important part of an integrated management of AAT [4]. Detecting and identifying trypanosome species in tsetse flies is fundamental to understanding the epidemiology of the associated diseases and to inform the establishment of efficient tsetse control programs aiming at reducing the prevalence of AAT in a particular area. That way, tsetse control programs can, for example, focus on localities where a high prevalence of the most virulent pathogens is found and use appropriate technologies depending on the vector species.

Little information exists about trypanosome biodiversity and distribution in Nigerian tsetse populations. Eleven species of tsetse were reported in Nigeria [5] infesting approximately 70 % of the country’s land mass [6]. Some of those species are major vectors of *T.vivax*, *T. brucei* subspp. and *T. congolense* subspp. [7, 8]. However, most of the available data rely on microscopy which has been shown to be far less sensitive and accurate than DNA-based detection and identification methods [9]. DNA-based methods have the advantage of being more sensitive and able to identify trypanosomes to the subspecies level and to detect mixed infections. Detailed information is required to better identify and characterize trypanosome-infested areas within the context of the eradication of AAT by the Pan African Tsetse and Trypanosomiasis Eradication Campaign (PATTEC).

Although all tsetse species are susceptible to trypanosomes infection, differences in susceptibility exist between species. In general, species from the *morsitans* group are considered more susceptible to trypanosome infection than species from the *palpalis* group. Within each species, a variety of factors intrinsic to the insect host can influence the competence of each individual fly to trypanosomes. For example, it has been shown that the susceptibility to trypanosomes is influenced by the sex or the age at the time of the first infected meal [10]. Studies describing the distribution of trypanosome species diversity among different tsetse species can bring insights about the vector competence of the different tsetse species highlighting non-random associations between tsetse and trypanosome species.

The present study is focused on the three main tsetse vector of AAT in Nigeria, namely *Glossina palpalis palpalis, Glossina tachinoides* and *Glossina morsitans submorsitans*. Individuals from these three species were collected in two localities (two species in one locality and the third species in the second locality) in central Nigeria. We then used PCR-based methods to detect, identify and determine the prevalence of trypanosomes in the tsetse collected. The main objectives of the study were to (i) evaluate the species diversity of trypanosomes causing AAT in northern Nigeria; and (ii) attempt to identify some ecological and biological factors responsible for potential differences in the trypanosome prevalence.

## Methods

### Sample collection and DNA purification

Tsetse fly samples were collected from Yankari National Park and Wuya areas between March and August 2012 using biconical traps. Yankari National Park is in the south-central part of Bauchi State (9.749°N, 10.499°E), in northeastern Nigeria. It is composed of savannah grassland with well-developed patches of woodland. Wuya is in Niger State (10.000°N, 6.167°E) located in north-western Nigeria. It is a mountainous area with wooded savannah vegetation. *Glossina palpalis palpalis* flies were caught in Wuya while *G. morsitans submorsitans* and *G. tachinoides* were captured in Yankari. Flies were killed by desiccation and stored as dry carcasses until further analysis. Samples are available upon request.

The sex of each fly was recorded and then ‘head plus proboscis’ (H + P) and abdomen (ABD) were cut from the thorax. Before tissue lysis, each ABD sample was cut into four pieces using a single-use scalpel blade. For each H + P, the sample was frozen in liquid N_2_ and then ground using a pestle (only the external part of the tube containing the sample was in contact with liquid N_2_, not the sample itself). DNA extraction and purification was carried out on all samples using the DNeasy blood and tissue kit (Qiagen, Manchester, UK) according to the manufacturer’s instruction for animal tissues except for the final elution volumes: one elution of 100 μl for ABD samples and two elutions of 35 and 30 μl for H + P (the two elutions were done in the same tube and a final volume of ~60 μl was obtained).

As trypanosome positive controls, we used purified DNA samples from parasites cryopreserved in mouse blood: *T. b. brucei* (strain STIB 247), *T. vivax* (ILRAD V-34), *T. congolense* Savannah (IL3000), *T. congolense* Forest (ANR3). DNA was purified from 100–300 μl cryostabilates with > 6.3 × 10^7^ trypanosomes/ml using the DNeasy blood and tissue kit (Qiagen, Manchester, UK) according to the manufacturer’s instructions for cultured cells.

### Trypanosome detection and identification by PCR

Detection and identification of trypanosomes were undertaken using the ITS1 PCR assay developed by Njiru et al. [9]. This PCR assay allows the detection and identification of *Trypanosoma vivax*, *T. simiae* Tsavo, *T. godfreyi*, *T. simiae*, *T. congolense* Kilifi and the subgenus *Trypanosoon* (*T. brucei* ssp. and *T. evansi*). Using this PCR assay, a single band at ~700 bp is obtained for both *T. congolense* Savannah and *T. congolense* Forest and species-specific PCRs are thus subsequently required to identify these trypanosomes at the subspecies level.

ITS1 PCR amplifications were carried out in 10 μl reaction mixture containing 5.9 μl distilled water, 1 μl of 10× Custom PCR Master Mix (45 mM Tris-HCl (pH 8.8), 11 mM (NH_4_)_2_SO_4_, 4.5 mM MgCl_2_, 0.113 mg/ml BSA, 4.4 μM EDTA, 1 mM each of dATP, dCTP, dGTP and dTTP) (Thermo Scientific, ABgene®UK), 1 μl of each primer solution at 10 μM (see Table 1 for primer sequences), 0.1 μl of Taq polymerase at 5 U/μl (Thermo Scientific, ABgene®UK) and 1 μl DNA template. The cycling conditions were as follows: (95 °C, 2 min); 30 cycles of (95 °C, 50 s), (60 °C, 50 s) and (72 °C, 1 min); (72 °C, 5 min). For each ITS1 PCR performed a negative control (distilled water) as well as positive controls for *T. brucei brucei*, *T. congolense* Savannah and *T. vivax* (see the previous section for details) were included. Gel electrophoreses were carried out using 5 μl of PCR product in 1.5 % agarose gels stained with ethidium bromide (5 μg/ml). PCR products were then visualized using UV and gels were photographed.

**Table 1:** PCR primers used

Target	Amplicon size (bp)	Forward primer name^b^	Forward primer sequence (5’-3’)	Reverse primer name^b^	Reverse primer sequence (5’-3’)	Reference
Trypanosome ITS1	250 to 700^a^	ITS1 CF	CCGGAAGTTCACCGATATTG	ITS1 BR	TTGCTGCGTTCTTCAACGAA	[9]
*T. congolense* Savannah-specific satellite DNA	316	TCS1	CGAGAACGGGCACTTTGCGA	TCS2	GGACAAACAAATCCCGCACA	[22]
*T. congolense* Forest-specific satellite DNA	350	TCF1	GGACACGCCAGAAGGTACTT	TCF2	GTTCTCGCACCAAATCCAAC	[22]

^a^Various sizes between 250 and 700 bp depending on the trypanosome(s) species present in the sample (*T. brucei* ssp.: ~480 bp; *T. congolense* Savannah/Forest: ~700 bp, *T. congolense* Kilifi ~620 bp; *T. simiae*: ~400 bp; *T. simiae* Tsavo: ~370 bp; *T. godfreyi*: ~300 bp and *T. vivax*: ~250 bp; ^b^Name used in the initial publication

Samples positive for *T. congolense* (ITS1 band at ~700 bp) were further screened using subspecies-specific PCR to determine the subspecies of *T. congolense* (Savannah or Forest) the fly was infected with. Subspecies-specific PCR primer pairs were used (see Table 1). For both subspecies, PCR amplifications were carried out in 10 μl reactions containing 1 μl of template DNA, 3 μl of distilled water, 1 μl of a primer mix (F + R) at 10 μM each and 5 μl of Dream Taq green Mater Mix (Thermo Scientific, UK). The cycling conditions were as follows: (95 °C, 3 min); 30 cycles of (95 °C, 30 s), (58 °C, 30 s) and (72 °C, 30 s); (72 °C, 5 min). For each *T. congolense* subspecies-specific PCR performed, a negative control (distilled water) as well as a positive control, for *T. congolense* Savannah or Forest (see the previous section for details), were included. Gel electrophoreses were carried out using 4.5 μl of PCR product in 1.5 % agarose gels stained with ethidium bromide (0.8 mg/ml). PCR products were then visualized using UV and gels were photographed.

### Statistics

The data for H + P and ABD samples were compared using McNemar’s test. We tested the effect of the following explanatory variables on trypanosome prevalence using multiple logistic regression analyses: ‘tsetse species’, ‘collection site’ and ‘fly sex’. For all multiple regressions analyses performed on trypanosome prevalence a fly was considered infected if either the ABD or the H + P sample was infected. Multiple logistic regressions were performed for “all trypanosomes”, *T. congolense* subspp. (which refers to the prevalence of both *T. congolense* subspecies combined), *T. congolense* Savannah, *T.* congolense Forest and *T. vivax*. The prevalence of other types of infection present only in one tsetse species (*T. simiae* and *T. godfreyi*) was considered as part of the category “all trypanosomes” rather than individually.

Because one tsetse species is only present in one of the sites (*G. p. palpalis* in Wuya) and the other two species are only present in the second site (*G. tachinoides* and *G. m. submorsitans* in Yankari) it was not possible to include ‘tsetse species’ and ‘collection site’ as explanatory variables in a multiple logistic regression applied to the complete dataset.

Thus, ‘collection site’, ‘fly sex’ and their interaction were included as explanatory variables in a multiple logistic regression applied to the complete dataset [prevalence ~ ‘collection site’ + ‘fly sex’ + (‘collection site’ × ‘fly sex﻿’)]. The association between the trypanosome prevalence and the tsetse species could only be investigated within the site of Yankari. ‘tsetse species’, ‘fly sex’ and their interaction were thus included as explanatory variables in a multiple logistic regression applied to the Yankari dataset [prevalence ~ ‘tsetse species’ + ‘fly sex’ + (‘tsetse species’ × ‘fly sex’)].

All statistical analyses were performed using R [11]. Multiple logistic regressions were performed using the *glm* function from the package *stats*. A binomial probability distribution was used and a logit link function was applied to the dependent variable, i.e. trypanosome prevalence. Backward model selection was performed using the *stepAIC* function from the MASS package [12] to identify the most relevant explanatory variables among the ones considered. The complete trypanosome prevalence dataset is provided in Additional file 1 and the R code used for the multiple logistic regression analyses is provided as Additional file 2.

## Results

The average apparent tsetse densities recorded during the trapping period (March-August 2012) were 27.31 flies/trap/day for *G. p. palpalis* in Wuya and 128.03 and 101.21 flies/trap/day for *G. tachinoides* and *G. m. submorsitans* in Yankari, respectively. We screened 488 tsetse flies of three species for the presence of trypanosomes using the ITS1 PCR approach developed by Njiru et al. [9]. All samples that were positive for *T. congolense* subspp. (PCR product at ~700 bp, *n* = 26) were then screened using subspecies-specific PCR to determine whether the fly was infected by *T. congolense* Savannah and/or by *T. congolense* Forest.

**Fig. 1:**
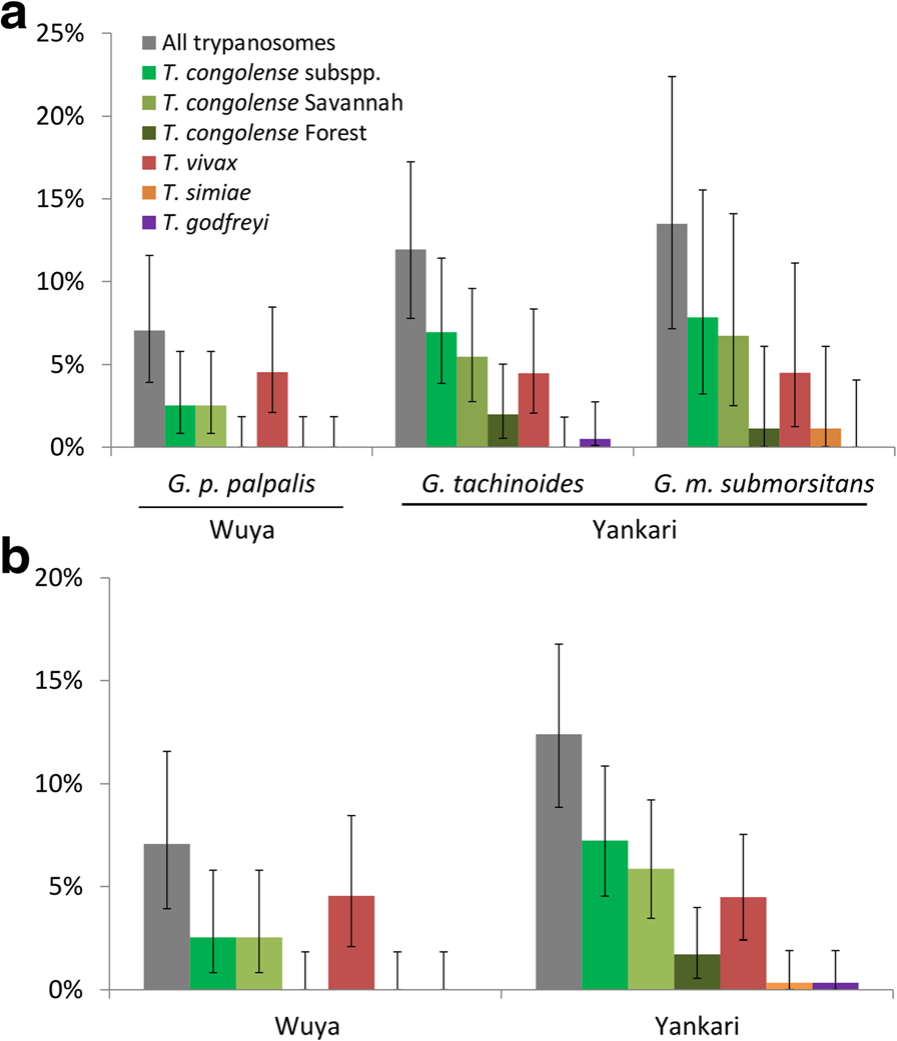
Trypanosome prevalence in the three species (**a**) and the two sites (**b**) studied. Error bars corresponds to 95 % confidence intervals

Trypanosome species diversity seemed higher in Yankari than in Wuya (five and two species, respectively; Table 2 and Fig. 1) with *T. simiae*, *T. congolense* Forest and *T. godfreyi* detected in Yankari at low prevalence but not detected in Wuya. It must be noted that the upper limits of the prevalence confidence intervals indicate that *T. simiae*, *T. congolense* Forest and *T. godfreyi* could be present in Wuya. The most prevalent trypanosomes species in both sites were *T. congolense* Savannah and *T. vivax* (Table 2 and Fig. 1)*.* Only one infection of *T. simiae* and *T. congolense* Forest were found in *G. m. submorsitans*. Similarly, only one infection of *T. godfreyi* and four infections of *T. congolense* Forest were detected in *G. tachinoides*. In *G. p. palpalis*, the tsetse species collected in Wuya, only *T. congolense* Savannah and *T. vivax* were detected (Table 2 and Fig. 1).

**Table 2:** Prevalence (95 % confidence intervals) of trypanosomes in male and female flies

Species	Sex	*n*	All	*T. c.* subspp.	*T. c.* S.	*T. c.* F.	*T. v.*	*T. s.*	*T. g.*
*G. p. palpalis*	Male	98	6.1 % (2.3–12.9 %)	3.1 % (0.6–8.7 %)	3.1 % (0.6–8.7 %)	0.0 % (0–3.7 %)	3.1 % (0.6–8.7 %)	0.0 % (0–3.7 %)	0.0 % (0–3.7 %)
	Female	100	8.0 % (3.5–15.2 %)	2.0 % (0.2–7.0 %)	2.0 % (0.2–7.0 %)	0.0 % (0–3.6 %)	6.0 % (2.2–12.6 %)	0.0 % (0–3.6 %)	0.0 % (0–3.6 %)
*G. tachinoides*	Male	40	2.5 % (0–13.2 %)	2.5 % (0–13.2 %)	2.5 % (0–13.2 %)	0.0 % (0–8.8 %)	0.0 % (0–8.8 %)	0.0 % (0–8.8 %)	0.0 % (0–8.8 %)
	Female	161	14.3 % (9.3–20.7 %)	8.1 % (4.4–13.4 %)	6.2 % (3–11.1 %)	2.5 % (0–6.2 %)	5.6 % (2.6–10.4 %)	0.0 % (0–2.3 %)	0.6 % (0–3.4 %)
*G. m. submorsitans*	Male	17	11.8 % (1.5–36.4 %)	5.9 % (0–28.7 %)	0.0 % (0–19.5 %)	5.9 % (0–28.7 %)	0.0 % (0–19.5 %)	5.9 % (0–28.7 %)	0.0 % (0–19.5 %)
	Female	72	13.9 % (6.9–24.1 %)	8.3 % (3.1–17.3 %)	8.3 % (3.1–17.3 %)	0.0 % (0-5 %)	5.6 % (1.5–13.6 %)	0.0 % (0–5 %)	0.0 % (0–5 %)

Notes: In agreement with *ISID* News, this table is a modification of the one available in the fellowship report by Issac et al. [23], * n*: sample size; All: All trypanosomes; *T. c.* subspp.*: Trypanosoma congolense* subspp. (which refers to both *T. congolense* subspecies combined); *T. c.* S.: *Trypanosoma congolense* Savannah; *T. c.* F.: *Trypanosoma congolense* Forest; *T. v.*: *Trypanosoma vivax*; *T. s.*: *Trypanosoma simiae*; *T. g.*: *Trypanosoma godfreyi*. 95 % confidence intervals are indicated in parentheses

To collate these data we screened DNA preparations from both ABD and H + P samples from more than 95 % of the flies (this could not be done for all the flies because some fly heads detached from their body during storage). Comparisons of the results obtained for each of these body parts are shown in Tables 3 and 4 for *T. congolense* subspp. and *T. vivax* respectively. For *T. congolense* subspp., there were very few flies that were positive in both ABD and H + P and most infections were found only in the abdomen samples (*χ*^2^ = 8.47, *df* = 1, *P* < 0.01). In contrast, *T. vivax* was detected only in the H + P samples (*χ*^2^ = 20.05, *df* = 1, *P* < 0.001). In *G. p. palpalis*, *T. congolense* Savannah was detected in some ABD and some H + P samples. In *G. tachinoides*, *T. congolense* Savannah and Forest were detected in some ABD and some H + P samples while *T. godfreyi* was detected in a single H + P sample. In *G. m. submorsitans*, *T. congolense* Savannah was detected in some ABD and some H + P samples while *T. congolense* Forest and *T. simiae* were only detected in ABD samples.

**Table 3:** *Trypanosoma congolense* subspp. detected using ITS1 PCR in abdomen and ‘head plus proboscis’ DNA preparations

		Abdomen	
		Positive	Negative
Head plus proboscis	Positive	6 (1.3 %)	2 (0.4 %)
	Negative	15 (3.2 %)	442 (95.1 %)

Note: Data are combined for all three species of tsetse fly. *Trypanosoma congolense* subspp. refers to both *T. congolense* subspecies combined

**Table 4:** *Trypanosoma vivax* detected using ITS1 PCR in abdomen and ‘head plus proboscis’ DNA preparations

		Abdomen	
		Positive	Negative
Head plus proboscis	Positive	0	22 (4.7 %)
	Negative	0	443 (95.3 %)

Note: Data are combined for all three species of tsetse fly

The differences in prevalence between male and female flies of each of the three species of tsetse are shown in Table 2 for each trypanosome species detected as well as the categories “All trypanosomes” and *T. congolense* subspp. To investigate the differences in prevalence between ‘tsetse species’, ‘collection site’ and ‘fly sex’ we undertook multiple logistic regression analyses, the outputs of which are summarised in Table 5. Infection prevalence of ‘All trypanosomes’ was higher in female than in male flies (Tables 2 and 5, *Z* = 2.165, *P* = 0.030). The prevalence of *T. congolense* subspp. was significantly higher in Yankari (considering both tsetse species together in this locality) than in Wuya (Fig. 1b, Table 5; *Z* = 2.185, *P* = 0.029). There were no statistically significant differences in trypanosome prevalence between *G. tachinoides* and *G. m. submorsitans* in Yankari (Fig. 1a, Table 5).

**Table 5:** Multiple logistic regression analyses on trypanosome prevalence

Dataset considered	Trypanosome prevalence investigated	Explanatory variables in the selected model	Odd ratio	*P*-value
On complete dataset	All trypanosomes	‘fly sex’	2.286	**0.030**
	*T. congolense* subspp.	‘collection site’	3.025	**0.029**
	*T. congolense* Savannah	‘collection site’	0.565	0.625
		‘fly sex’	0.646	0.637
		‘collection site’ × ‘fly sex’	6.419	0.182
	*T. congolense* Forest	‘collection site’	4.1 × 10^7^	0.993
	*T. vivax*	‘collection site’	2.7 × 10^-7^	0.986
		‘fly sex’	2.021	0.330
		‘collection site’ × ‘fly sex’	3.4 × 10^6^	0.986
On Yankari dataset	All trypanosomes	‘fly sex’	2.985	0.079
	*T. congolense* subspp.	‘fly sex’	2.453	0.237
	*T. congolense* Savannah	‘fly sex’	4.148	0.172
	*T. congolense* Forest	‘tsetse species’	5.3 × 10^7^	0.995
		‘fly sex’	2.2 × 10^7^	0.995
		‘tsetse species’ × ‘fly sex’	8.6 × 10^-16^	0.992
	*T. vivax*	‘fly sex’	1.9 × 10^7^	0.991

Note: For the complete dataset the complete model considered before model selection was: prevalence ~ ‘collection site’ + ‘fly sex’ + (‘collection site’ × ‘fly sex’). For the Yankari dataset the complete model considered before model selection was: prevalence ~ ‘tsetse species’ + ‘fly sex’ + (‘tsetse species’ × ‘fly sex’). ×: indicates the interaction between two explanatory variables. *Trypanosoma congolense* subspp. refers to both *T. congolense* subspecies combined. *P*-value: *P*-value associated with each explanatory variable. Significant results are shown in bold

## Discussion

### Identification of major pathogens causing AAT

Our results show that tsetse flies in the two study sites harbour mature infections of *T. vivax*, *T. congolense* Savannah, *T. congolense* Forest and *T. godfreyi*. This implies that there is an active transmission of these trypanosomes in the two sites studied. Among the trypanosome species associated with mature infections in tsetse, *T. vivax* and *T. congolense* Savannah are likely to be the main parasites responsible for African Animal Trypanosomiasis (AAT) in livestock. *T. vivax* was only detected in the H + P samples. This is expected since this parasite only develops in the mouthparts [13] although it is possible to detect *T. vivax* in the abdomen if the fly has recently taken an infected meal. These results are in agreement with a recent survey carried out in cattle in northern Nigeria to discriminate *T. congolense* subspecies using molecular tools [14]. The presence of both subspecies of *T. congolense* is of importance for the management of AAT in Nigeria because *T. congolense* Savannah is more virulent than *T. congolense* Forest [15]. Indeed, the management of the disease caused by each *T. congolense* subspecies would likely be more effective if it could be subspecies-specific.

### Trypanosome diversity and prevalence are different between sites

Although the upper limits of the prevalence confidence intervals suggest that *T. simiae*, *T. congolense* Forest and *T. godfreyi* could be present in Wuya, trypanosome species diversity seemed higher in Yankari (five species) than in Wuya (two species). In Wuya, only the two main pathogens responsible for AAT (*T. congolense* Savannah and *T. vivax*) were detected. This probably reflects the fact that tsetse in the Yankari National Park have a more diverse host range, feeding on wild animals, than the tsetse from Wuya that are likely feeding on a restricted number of livestock species. Indeed, the availability of hosts can influence trypanosome infection rates in tsetse [16]. Yankari game reserve is home to both wild and domestic animals while only domestic animals are seen in Wuya.

Combining the data for both species in Yankari, we were able to identify a difference between sites with the prevalence of *T. congolense* subspp. being higher in Yankari than in Wuya. The fact that the prevalence of *T. congolense* subspp. is higher in flies from Yankari than in flies from Wuya is probably a consequence of the higher prevalence of *T. congolense* subspp. in wild tsetse hosts in Yankari than in domesticated animals in Wuya. A complementary or alternative hypothesis would be that tsetse flies from Yankari are more susceptible to *T. congolense* subspp. than flies from Wuya. Our sampling design does not allow testing of that hypothesis because we could not sample the same species in both sites.

In Yankari, there were no statistically significant differences in trypanosome prevalence between *G. tachinoides* and *G. m. submorsitans*. This is a particularly interesting result because it goes against the traditional view that tsetse from the *morsitans* group are considered to be more susceptible to trypanosomes than tsetse from the *palpalis* group. The data generally gathered to support this concept have often been collected using approaches where the ‘species effect’ is confounded with a potential geographical effect. Indeed, experimental approaches have generally used tsetse laboratory colonies originating from different regions e.g. [17] and field studies often compared trypanosome prevalence of allopatric species. This is not the case in the present study as *G. m. submorsitans* and *G. tachinoides* are sympatric in Yankari. However, one cannot make conclusions on tsetse susceptibility based on data collected at a single field site. It must be noted that some experimental data have also shown similar vectorial capacity between tsetse from the *morsitans* and the *palpalis* group e.g. [11].

### Trypanosome prevalence is higher in females than males

Our results clearly show that the trypanosome prevalence is higher in the female than the male flies analysed. Although this effect is likely mainly driven by a stronger effect for *T. vivax* infections, it must be noted that it was also the case for other trypanosome species (*T. congolense* Savannah in *G. m. submorsitans* and *T. congolense* Savannah and Forest in *G. tachinoides*). Although experimental data do not support significant differences in trypanosome infections between male and female tsetse [18, 19], higher trypanosome prevalence in females than in males have been previously reported in the field e.g. [20]. Such a result could be explained by the fact that female tsetse flies live longer than males. Females are thus more likely than males to be exposed to trypanosome infection during their lifetime. In line with that assertion, a study has shown that the prevalence of *T. vivax* rises approximately linearly with fly age [21]. The higher trypanosome prevalence observed in females would thus imply that the collected females were on average older than the collected males. Such a hypothesis could thus be tested in the future by estimating the age of the flies collected.

## Conclusions

Our results show that *T. congolense* Savannah and *T. vivax* are the main species of parasite potentially causing AAT in the two study sites and that Yankari National Park is a potential reservoir of trypanosomes both in terms of parasite abundance and species diversity. This study constitutes the first report of *T. congolense* Savannah and *T. congolense* Forest in tsetse in Nigeria; *T. congolense* Savannah being more prevalent than *T. congolense* Forest. The presence of both subspecies and the difference in their prevalences are of importance for the management of AAT in Nigeria because *T. congolense* Savannah is more virulent than *T. congolense* Forest [15].

## Additional files

Additional file 1: Complete trypanosome prevalence dataset: file “Isaac_et_al_additional_file_1_complete_trypanosome_prevalence_dataset.txt”. (TXT 13 kb) Additional file 2: R code used for the multiple logistic regression analyses: file “Isaac_et_al_additional_file_2_R_code_for_multiple_logistic_regressions.txt”. (TXT 7 kb)
